# Senescent cancer cell-derived nanovesicle as a personalized therapeutic cancer vaccine

**DOI:** 10.1038/s12276-023-00951-z

**Published:** 2023-03-01

**Authors:** Jihye Hong, Mungyo Jung, Cheesue Kim, Mikyung Kang, Seokhyeong Go, Heesu Sohn, Sangjun Moon, Sungpil Kwon, Seuk Young Song, Byung-Soo Kim

**Affiliations:** 1 Interdisciplinary Program for Bioengineering, Seoul National University, Seoul, 08826 Republic of Korea; 2 School of Chemical and Biological Engineering, Seoul National University, Seoul, 08826 Republic of Korea; 3 Institute of Chemical Processes, Institute of Engineering Research, BioMAX, Seoul National University, Seoul, 08826 Republic of Korea

**Keywords:** Nanoparticles, Vaccines

## Abstract

The development of therapeutic cancer vaccines (TCVs) that provide clinical benefits is challenging mainly due to difficulties in identifying immunogenic tumor antigens and effectively inducing antitumor immunity. Furthermore, there is an urgent need for personalized TCVs because only a limited number of tumor antigens are shared among cancer patients. Several autologous nanovaccines that do not require the identification of immunogenic tumor antigens have been proposed as personalized TCVs. However, these nanovaccines generally require exogenous adjuvants (e.g., Toll-like receptor agonists) to improve vaccine immunogenicity, which raises safety concerns. Here, we present senescent cancer cell-derived nanovesicle (SCCNV) as a personalized TCV that provides patient-specific tumor antigens and improved vaccine immunogenicity without the use of exogenous adjuvants. SCCNVs are prepared by inducing senescence in cancer cells ex vivo and subsequently extruding the senescent cancer cells through nanoporous membranes. In the clinical setting, SCCNVs can be prepared from autologous cancer cells from the blood of liquid tumor patients or from tumors surgically removed from solid cancer patients. SCCNVs also contain interferon-γ and tumor necrosis factor-α, which are expressed during senescence. These endogenous cytokines act as adjuvants and enhance vaccine immunogenicity, avoiding the need for exogenous adjuvants. Intradermally injected SCCNVs effectively activate dendritic cells and tumor-specific T cells and inhibit primary and metastatic tumor growth and tumor recurrence. SCCNV therapy showed an efficacy similar to that of immune checkpoint blockade (ICB) therapy and synergized with ICB. SCCNVs, which can be prepared using a simple and facile procedure, show potential as personalized TCVs.

## Cancer: Turning tumors into vaccines

Therapeutic vaccines derived from tumor cells could provide a personalized strategy for training the immune system to find and destroy cancer. Past efforts to develop such vaccines have been hampered by the challenge of identifying tumor-specific proteins that elicit a strong immune response. To overcome this, Byung-Soo Kim and colleagues at Seoul National University pursued a strategy in which they subjected tumor cells to chemical treatments that halted proliferation and stimulated production of immunity-activating signaling molecules. The cells were then broken up into membrane-encased fragments filled with tumor-derived biomolecules. Mice dosed with such vaccines, which carry a diverse mix of antigens, showed a potent anti-cancer response, resulting in tumor cell death and significantly improved survival. By treating cells from patient biopsies in this fashion, clinicians could potentially derive potent, individually tailored tumor vaccines.

## Introduction

Cancer immunotherapies, such as immune checkpoint blockade therapy and chimeric antigen receptor T (CAR-T) cell therapy, activate antitumor immunity in cancer patients and have shown significant success in clinical oncology^1–4^. Therapeutic cancer vaccines (TCVs), another cancer immunotherapy approach, involve the administration of immunogenic tumor antigens to stimulate the patient adaptive immune system against the tumor^5^. However, the development of clinically applicable TCVs has been limited thus far. A tumor antigen-loaded DC-based TCV (Sipuleucel-T) was approved by the FDA in 2010 to treat prostate cancer^6^, but no further TCVs have been approved. The difficulties associated with the identification of immunogenic tumor antigens and insufficient antitumor immunity of TCVs are the main limitations in the development of clinically applicable TCVs^7^.

A key step in the TCV development process is the identification of immunogenic tumor antigens that elicit antitumor immunity. Various tumor-associated antigens (TAAs) have been suggested as new target antigens for TCVs, including human epidermal growth factor receptor-2 (HER-2) and mesothelin and melanoma-associated antigen recognized by T cells (MART-1)^8,9^. Recently, neoantigens, which are nonself antigens that are expressed in only mutated cancer cells, have emerged as new target antigens for TCVs. Neoantigens can be profiled by next-generation sequencing-based cancer exome sequencing^10,11^. However, most TCVs employing TAAs or neoantigens have failed to achieve therapeutic benefit in clinical trials^12,13^.

The disappointing clinical results for TCVs employing TAAs are mainly due to the nonexclusive and heterogeneous expression of TAAs in tumor tissues^10^. TAAs are also expressed in normal tissues, resulting in off-target side effects mediated by activated T cells or elimination of TAA-specific T cells through immune tolerance. In addition, the heterogeneous expression of TAAs in tumors leads to low effectiveness in the vaccine-mediated killing of tumors.

Meanwhile, neoantigens are highly individual specific, and only a small number of neoantigens are shared among cancer patients^10^. This demands the development of personalized TCVs. Recently, next-generation sequencing technology and whole-genome mapping have made it feasible to identify patient-specific neoantigens and design personalized TCVs^14^. However, most of the discovered neoantigens exhibit low immunogenicity or low affinity for major histocompatibility complex (MHC) molecules. Profiling of cancer patients has revealed that only a small fraction (~1–2%) of neoantigens in cancer cells are recognized by T cells and induce sufficient immune responses^15^. In addition, some neoantigens often disappear in tumor tissues due to the rapid mutation rate in tumor cells. This poses a problem for the development of patient-specific neoantigen TCVs.

Nanovaccines made of autologous cancer cell membranes have been proposed as personalized TCVs that do not require the identification of immunogenic tumor antigens^16,17^. However, inducing sufficient antitumor immune responses in vivo often requires the addition of an appropriate exogenous adjuvant that induces dendritic cell (DC) activation^18^, and nanovaccines made of autologous cancer cell membranes generally require exogenous adjuvants (e.g., TLR agonists) to improve vaccine immunogenicity. These adjuvants include CpG oligodeoxynucleotides (CpG ODNs), resiquimod (R848), and polyinosinic:polycytidylic acid (poly I:C)^19,20^. However, these TLR agonists raise safety concerns due to the potential to cause severe adverse effects^21,22^. Thus, the use of safe and effective adjuvants is required to achieve safe and effective TCV therapy.

**Fig. 1:**
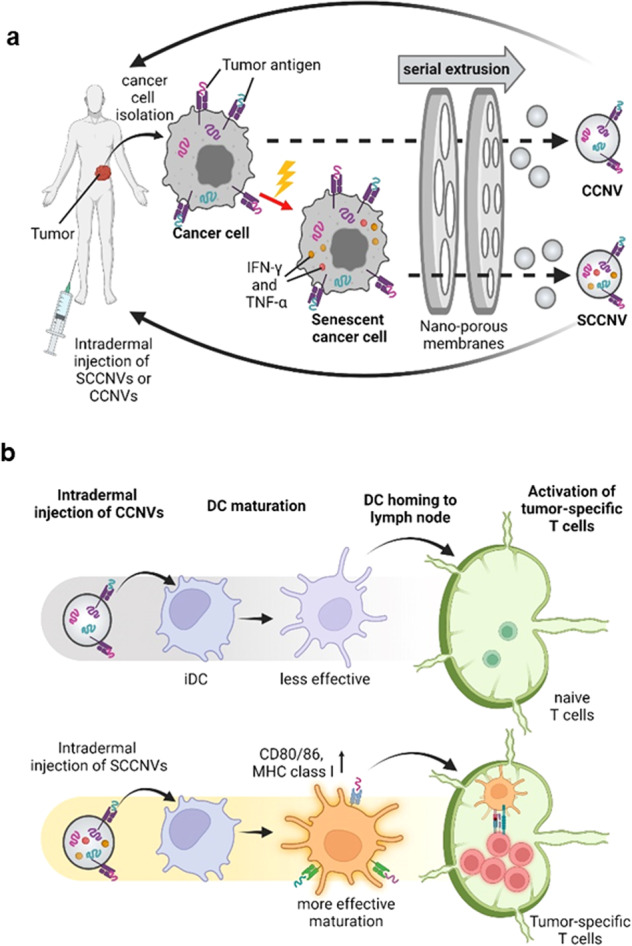
Experimental scheme of SCCNVs. Illustration of (**a**) SCCNV preparation and personalized cancer vaccination and (**b**) the proposed mode of action of the vaccines. SCCNV senescent cancer cell-derived nanovesicle, CCNV cancer cell-derived nanovesicle, iDC immature dendritic cell, mDC mature dendritic cell.

Here, we present senescence-induced cancer cell-derived nanovesicle (SCCNV) as a personalized TCV that can overcome the limitations of current TCVs. Nanovesicles are exosome-mimetic nanosized vesicles produced by extruding cells through nanoporous membranes^23^. Nanovesicles can deliver RNAs and proteins originating from parental cells and exhibit a higher production efficiency than naturally secreted exosomes^23,24^. Nanovesicles derived from various immune cells, including T cells, macrophages and dendritic cells, have been explored for cancer immunotherapy^24–26^. SCCNVs were produced by serial extrusion of senescence-induced cancer cells (Fig. 1a). Autologous cancer cells for SCCNV preparation would be clinically available from cancer patients, and SCCNVs prepared from autologous cancer cells can deliver a variety of patient-specific tumor antigens, avoiding the complicated process of neoantigen identification. While TCVs with a single TAA or neoantigen may face immune escape that is caused by the ceaseless mutation of cancer cells in vivo, SCCNVs that would contain a spectrum of tumor antigens may avoid the immune escape problem. Cellular senescence is a phenomenon characterized by arrest during cell division in response to various cellular stresses, such as DNA damage and oxidative stress^27^. Senescent cells exhibit a senescence-associated secretory phenotype (SASP), which includes expression of interferon-gamma (IFN-γ) and tumor necrosis factor-alpha (TNF-α)^28^. In the present study, IFN-γ and TNF-α were expressed during ex vivo senescence induction and contained in SCCNVs. These endogenous cytokines served as adjuvants that enhance the immunogenicity of the vaccine. These endogenous adjuvants would be safer than conventional exogenous adjuvants, such as MPLA, poly I:C and CpG, which may have safety concerns^21,22^. Intradermally injected SCCNVs could deliver tumor antigens and adjuvants (IFN-γ and TNF-α) to DCs, resulting in DC activation, DC migration to the draining lymph nodes, and activation of tumor-specific T cells capable of cancer cell killing (Fig. 1b).

## Experimental section

### Experimental animals

All animal experiments were approved by the Institutional Animal Care and Use Committee (IACUC) of Seoul National University (SNU-200630-2-4) and performed in compliance with the guidelines of the IACUC. For in vivo studies, six-week-old C57BL/6 mice and six-week-old B/C mice were purchased from Orient Bio (Gyeonggi, Korea) or JA Bio (Gyeonggi, Korea).

### Cell culture

B16F10 cells were purchased from American Type Culture Collection (ATCC; VA, USA) and were cultured in Dulbecco’s modified Eagle’s medium (DMEM; Gibco, NY, USA) supplemented with 10% (v/v) FBS and 1% (v/v) PS. E.G7-OVA cells, a variant of the EL4 cell line that expresses full-length OVA, were a gift from Professor Junsang Doh, Seoul National University, Seoul, Korea. E.G7-OVA cells were cultured in RPMI 1640 medium (Gibco) containing 10% (v/v) FBS, 1% (v/v) PS, 10 mM HEPES, 1.0 mM sodium pyruvate, 0.05 mM β-mercaptoethanol and 0.04 μg/ml G-418 antibiotics (Roche). 4T1 cells were purchased from ATCC and cultured in RPMI 1640 medium containing 10% (v/v) FBS and 1% (v/v) PS. Primary T cells were isolated from the spleen of 6-week-old female C57BL/6 mice (Orient Bio, Gyeonggi, Korea) using the MojoSort™ Mouse CD8 T Cell Isolation Kit (BioLegend) according to the manufacturer’s protocol. Cells were then cultured in RPMI 1640 medium supplemented with 10% (v/v) FBS, 1% (v/v) PS, 1% (v/v) GlutaMAX (Gibco), 10 mM HEPES, 1 mM sodium pyruvate and 55 μM 2-mercaptoethanol.

### Preparation of SCCNVs

SCCNVs were produced from cancer cell lines. For senescence induction, 0.1 μM doxorubicin hydrochloride (doxorubicin HCl; Sigma–Aldrich, MO, USA) was added to the cell culture medium for 24 h. Then, the cells were washed with PBS, changed to doxorubicin-free medium and cultured for an additional 4 days^29,30^. Senescence-induced cancer cells were detached from the cell culture plate using trypsin-EDTA, washed with PBS and serially extruded through polycarbonate membrane filters (Whatman, UK) with pore sizes of 1 μm, 400 nm and 200 nm using a mini-extruder (Avanti Polar Lipids, AL, USA) to obtain nanovesicles as previously reported^23^. To reload antigen peptides that might have been dissociated from the MHC class I molecules on nanovesicles onto the MHC class I molecules, the pH of the nanovesicle-containing solution was adjusted to 5.5 with sodium acetate buffer for 30 min and then neutralized with Tris-HCl buffer^31,32^. The nanovesicles were then centrifuged at 30,000 × *g* for 1 h at 4 °C. The protein concentration of isolated SCCNVs was quantified using Bradford reagent (Sigma–Aldrich) according to the manufacturer’s protocol.

### Optimization of senescence induction

B16F10 cells were treated with doxorubicin HCl at concentrations of 0, 0.1, 0.5, and 1 μM. To evaluate senescence, the cells were stained with a senescence-associated β-galactosidase (SA-β-gal) staining kit (Cell Signaling Technology, MA, USA) according to the manufacturer’s protocol. To evaluate the cytotoxicity of doxorubicin HCl, Cell Counting Kit-8 (CCK-8; DoGenBio, Seoul, Korea) was used according to the manufacturer’s protocol.

### Characterization of SCCNVs

The size distributions of SCCNVs were assessed using dynamic light scattering (Zetasizer Nano ZS, Malvern Panalytical, UK). To evaluate the colloidal stability of SCCNVs in a 30% FBS-containing buffer, the hydrodynamic diameter of SCCNVs was detected with a Zetasizer at various time points. The morphology of SCCNVs was evaluated with a JEM-2100 transmission electron microscope (JEOL, Japan) installed at the National Center for Inter-university Research Facilities (NCIRF) at Seoul National University. The relative mRNA expression levels of IFN-γ and TNF-α were determined with qRT–PCR analysis. mRNA was extracted from cancer cells or nanovesicles using QIAzol Lysis Reagent (Qiagen, CA, USA). RNA from each group was used for cDNA synthesis using PCR PreMix (Bioneer, Daejeon, Korea). SYBR green-based qRT–PCR was performed with TOPreal™ qPCR 2X PreMIX (Enzynomics, Daejeon, Korea). The cycling conditions were as follows: initial denaturation at 95 °C for 15 min, followed by 60 cycles at 95 °C for 10 s, 60 °C for 15 s and 72 °C for 30 s. The expression of each gene was normalized to that of GAPDH. The protein levels of IFN-γ and TNF-α were evaluated by western blotting using an anti-mouse IFN-γ antibody, anti-mouse TNF-α antibody (Bioss, MA, USA) and anti-mouse GAPDH antibody (Invitrogen, CA, USA). GAPDH was used as the control protein. For Coomassie blue staining, the proteins in lysates generated from normal B16F10 cells, senescent B16F10 cells, CCNVs and SCCNVs were separated using SDS–PAGE, and the polyacrylamide gel was stained with Coomassie blue using PageBlue™ Protein Staining Solution (Thermo Scientific, MA, USA) to visualize the proteins. The amounts of IFN-γ and TNF-α were detected by analyzing lysates generated from CCNVs and SCCNVs with an ELISA kit (BioLegend). The calculated amounts of IFN-γ and TNF-α were divided by the total protein amount to obtain the amounts of IFN-γ and TNF-α per 1 µg of SCCNVs.

### Isolation of BMDCs

BMDCs were isolated as previously described^33^. Briefly, 6-week-old C57BL/6 mice were sacrificed, and the femurs were isolated from the hind limbs. The bones were flushed with PBS using syringes to isolate bone marrow cells. Red blood cell lysis buffer was added. After centrifugation, mononuclear bone marrow cells were cultured in dishes containing 10 mL of differentiation medium consisting of RPMI 1640 medium supplemented with 20 ng/mL GM-CSF (R&D Systems, MN, USA) and 10% (v/v) FBS. After 3 days, 5 mL of fresh differentiation medium was added to the dishes. Differentiated BMDCs were collected on Day 10.

### In vitro DC maturation

For in vitro DC maturation analysis, 5 × 10^5^ BMDCs were plated in each well of 6-well plates prior to SCCNV treatment. To investigate SCCNV uptake by DCs, 20 µg of CCNVs or SCCNVs was stained with 1,1'-dioctadecyl-3,3,3',3'-tetramethylindocarbocyanine perchlorate (DiI, Invitrogen) according to the manufacturer’s protocol. BMDCs were treated with DiI-stained CCNVs or SCCNVs and analyzed by FACS using a BD Canto-II flow cytometer (BD Sciences, CA, USA). To determine mRNA levels in DCs, BMDCs were treated with 20 µg of CCNVs or SCCNVs for 4 h. Treatment with 100 ng/ml lipopolysaccharide (LPS; Sigma–Aldrich, MO, USA) was used as the positive control. Then, the cells were lysed with QIAzol lysis reagent (Qiagen, CA, USA) for mRNA extraction. The relative mRNA levels of IL-6, IL-12p40 and CCR7 were evaluated by qRT–PCR. The expression of each gene was normalized to that of GAPDH. For detection of surface protein expression on BMDCs, BMDCs were treated with 20 μg of CCNVs or SCCNVs for 24 h. Then, the BMDCs were detached with trypsin-EDTA and stained with the following antibodies: APC-Cy7 anti-mouse CD11c antibody, PE anti-mouse CD80 antibody, APC anti-mouse CD86 antibody and FITC anti-mouse MHC class I antibody (BioLegend, CA, USA). Fluorescently labeled DCs were analyzed with a BD Canto-II flow cytometer (BD Sciences). FACS data were analyzed using FlowJo software (TreeStar Inc., OR, USA). For proinflammatory cytokine-induced maturation of DCs, BMDCs were treated with CCNVs and recombinant TNF-α (100 ng/ml) and IFN-γ (20 ng/ml) (BioLegend)^34,35^. Then, the BMDCs were detached and stained with the following antibodies: APC-Cy7 anti-mouse CD11c antibody, PE anti-mouse CD80 antibody and APC anti-mouse CD86 antibody (BioLegend). For TNF-α and IFN-γ knockdown, predesigned siRNA Silencer Select siRNA was purchased from Ambion Invitrogen, and siRNA transfection was conducted with Lipofectamine™ RNAiMAX Transfection Reagent (Thermo Fisher) according to the manufacturer’s protocol. Briefly, senescence was induced by doxorubicin treatment, and 24 h later, the B16F10 cells were washed with fresh medium, and siRNA was transfected on Day 1, 2, or 3 for optimization. After 3 days of culture, the cells were harvested, and the mRNA and protein levels of TNF-α and IFN-γ were tested by qRT‒PCR and western blotting. Then, senescent B16F10 cells with TNF-α and IFN-γ knocked down were used to produce siRNA-transfected SCCNVs.

### In vivo imaging of SCCNVs

For in vivo imaging of intradermally injected nanovesicles, CCNVs or SCCNVs were stained with VivoTrack 680 (Perkin Elmer, MA, USA) according to the manufacturer’s protocol. VivoTrack-stained CCNVs or SCCNVs (20 μg) were suspended in 50 μl of PBS and intradermally injected into the right flank of mice (*n* = 4 animals). To monitor the biodistribution of the injected SCCNVs, the mice were sacrificed 24 h after the injection. Five major organs (the heart, lungs, liver, kidneys and spleen) and the lymph nodes near the injection site were retrieved. Fluorescence signals were acquired using IVIS spectrum computed tomography (Perkin Elmer) at a 680-nm excitation wavelength and quantified using Living Image 3.1 software. The fluorescence intensity of each organ or lymph node was normalized to the sum of the intensities of the organs and tumors in each group.

### In vivo DC maturation

For analysis of in vivo DC maturation, PBS, CCNVs or SCCNVs were intradermally injected into the right flank of 6-week-old C57BL/6 mice. Three days after the injection, the right inguinal lymph node was harvested from the mice, minced, and passed through a 70-μm pore filter. The separated single cells were stained with the following antibodies: PE-conjugated anti-mouse CD11c, APC-conjugated anti-mouse CD86 and FITC-conjugated anti-mouse MHC class I (BioLegend). Fluorescently labeled cells were analyzed with a BD Canto-II flow cytometer (BD Sciences). FACS data were analyzed using FlowJo software (TreeStar Inc.). To investigate DC accumulation in the lymph nodes after intradermal injection of PBS, CCNVs or SCCNVs, the right-side inguinal lymph nodes were harvested 7 days after the intradermal injection. Then, the lymph nodes were minced and passed through a 70-μm pore filter. The separated single cells were stained with a PE-conjugated anti-mouse CD11c antibody, and the number of DCs was obtained through FACS analysis.

### In vitro T-cell proliferation analysis

OVA-specific CD8^+^ T cells harvested from transgenic OT-1 mice were used for the in vitro T-cell proliferation assay. Briefly, the lymph nodes and spleen of OT-1 mice were harvested, minced, and passed through a 70-μm pore filter. Then, CD8^+^ T cells were isolated using a MojoSort CD8^+^ T-cell isolation kit (BioLegend) according to the manufacturer’s protocol. The isolated OT-1 CD8^+^ T cells were stained with a CFSE cell division tracker kit (BioLegend) and cocultured with splenocytes (including DCs) isolated from wild-type C57BL/6 mice. Then, 1 μg/ml CCNVs and SCCNVs, which were produced from EL4 cancer cells or OVA-expressing E. G7-OVA cancer cells, was added to the cultures. An OVA epitope peptide (257-264, 1 mg/ml; ANASPEC, CA, USA) was used as the positive control. After 3 days of culture, the cells were harvested and stained with a BV421-conjugated anti-mouse CD3 antibody and PE-Cy7-conjugated anti-mouse CD8 antibody (BioLegend). Then, the percentage of CFSE^low^ CD3^+^ CD8^+^ T cells was calculated.

### Mouse immunization model

To investigate the immune responses induced by SCCNVs, C57BL/6 mice were intradermally injected with PBS, CCNVs or SCCNVs three times every 6 days. Three days after injection, blood was harvested from the immunized mice through retro-orbital bleeding, and red blood cells and lymphocytes were collected by centrifugation at 3000 × *g* for 30 min. Then, the red blood cells were lysed using RBC lysis buffer (Gibco). The lymphocytes were then stained with a BV421-conjugated anti-mouse CD3 antibody, an APC-conjugated anti-mouse CD4 antibody and a PE-Cy7-conjugated anti-mouse CD8 antibody (BioLegend) and analyzed with a BD Canto-II flow cytometer (BD Sciences). Six days after the last immunization, splenocytes were harvested from the mice and restimulated with 1 μg/ml gp100 peptides, the antigenic epitope of B16F10 cancer cells, as described in previous studies^26^. After 3 days of culture, the CD8^+^ T cells in the splenocytes were stained with a BV421-conjugated anti-mouse CD3 antibody, an APC-conjugated anti-mouse CD4 antibody and a PE-Cy7-conjugated anti-mouse CD8 antibody (BioLegend), and the culture supernatants were analyzed with IFN-γ and TNF-α ELISA kits (BioLegend). For CFSE staining, splenocytes were stained with CFSE first and then restimulated with gp100 peptides. After 3 days, the splenocytes were stained with an anti-mouse CD3 antibody and a PE-Cy7-conjugated anti-mouse CD8 antibody (BioLegend) and analyzed on a BD Canto-II to detect CFSE^low^ CD3 + CD8 + T cells. For ELISpot analysis, an ELISpot Plus: Mouse IFN-γ (ALP) (MabTech, Sweden) kit was used to analyze antigen-specific T cells in splenocytes. Harvested splenocytes were restimulated with gp100 peptides and cultured in ELISpot plates, and IFN-γ spots were counted after 24 h of culture.

### In vivo prophylactic model

Six-week-old C57BL/6 mice were randomly divided into three groups, anesthetized with xylazine (10 mg/kg) and ketamine (100 mg/kg) and intradermally injected with PBS, CCNVs or SCCNVs (20 μg of nanovesicles in 50 μl of PBS) once a week for three weeks. Six days after the last immunization, the mice were subcutaneously injected with B16F10 cancer cells (5 × 10^5^ cells in 100 μL of PBS per mouse) in the right flank. Tumor size was measured every 3 days using a digital caliper and computed according to the ellipsoidal calculation formula: *V* = 0.5 × (longest diameter) × (shortest diameter). Mouse survival was monitored for 40 days. The mice bearing tumors exceeding 2,500 mm^3^ in size were euthanized with CO_2_ inhalation.

### In vivo tumor challenge model

Six-week-old C57BL/6 mice were anesthetized with rumpun (10 mg/kg) and ketamine (100 mg/kg) and subcutaneously injected with B16F10 cancer cells (5 × 10^5^ cells in 100 μL of PBS per mouse) in the right flank. On Days 5, 8, and 11, the mice were intradermally injected with PBS, CCNVs or SCCNVs (20 μg of nanovesicles in 50 μl of PBS). Tumor size was measured every 3 days using a digital caliper and computed according to the ellipsoidal calculation formula: *V* = 0.5 × (longest diameter) × (shortest diameter). Mouse survival was monitored for 30 days. The mice bearing tumors exceeding 2500 mm^3^ in size were euthanized with CO_2_ inhalation.

### Tumor-infiltrating lymphocyte analysis

Tumor-infiltrating lymphocytes were analyzed as previously described^36,37^. Briefly, 4 days after the last injection, the tumor masses were harvested from euthanized mice and weighed. Then, the tumor tissues were minced and passed through a 70-μm pore filter. The separated single cells were stained with the following antibodies: anti-mouse CD3, anti-mouse CD4, anti-mouse CD8, anti-mouse Foxp3, anti-mouse IFN-γ and anti-mouse TNF-α (BioLegend). Intracellular staining of tumor-infiltrating lymphocytes was conducted according to the manufacturer’s protocol. The staining results were analyzed using FlowJo software (TreeStar Inc.).

### Immunohistochemical staining of tumor sections

Tumor tissues were fixed in 4% PFA and kept in a 30% sucrose solution for 1 day. The fixed tissues were embedded in OCT (Scigen Scientific, CA, USA) and stored at -80 °C. The cryopreserved tissues were sectioned at a thickness of 10 μm using a cryostat microtome (Leica, Germany). For Foxp3 staining, tissue sections were washed with PBS twice, blocked, and permeabilized with 0.6% Triton X-100 and 10% donkey serum (Gibco) in PBS for 2 h. Then, the sections were incubated with anti-mouse Foxp3 antibodies (BioLegend) overnight at 4 °C. Any unbound antibodies were removed, and the cell nuclei were stained with DAPI for 10 min. Fluorescence images were obtained with an LSM 710 confocal microscope (Carl Zeiss). For terminal deoxynucleotidyl transferase-mediated dUTP nick end labeling (TUNEL) staining assays, tumor tissue sections were washed and stained using the DeadEnd™ Fluorometric TUNEL System (Promega, WI, USA) according to the manufacturer’s protocol. Images were obtained with an LSM 710 (Carl Zeiss), and the TUNEL-positive cell percentage was calculated using NIH ImageJ software (Bethesda).

### In vivo toxicity of SCCNVs

C57BL/6 mice were anesthetized with rumpun (10 mg/kg) and ketamine (100 mg/kg) and intradermally injected with PBS, CCNVs or SCCNVs (20 μg of nanovesicles in 50 μl of PBS). Blood samples were obtained at various time points. Serum was obtained from the blood samples by centrifugation at 3000 × *g* for 30 min. The levels of AST, ALT, creatinine and BUN in the serum were determined with a DRI-CHEM 3500 S chemical analyzer (Fujifilm, Japan). For histological analysis, major organs (the liver, lungs, spleen, heart and kidneys) were harvested 14 days after the first injection. The tissues were fixed in 4% PFA overnight at 4 °C and dehydrated in a 30% sucrose solution. The tissues were embedded in OCT compound (Scigen Scientific) and sectioned at a thickness of 10 μm using a cryostat microtome (Leica, Germany). The sections were stained with hematoxylin (Cancer Diagnostics, NC, USA) and eosin (BBC Biochemical, WA, USA) and imaged using an optical microscope (Olympus, Tokyo, Japan).

### Lung metastasis tumor model

Six-week-old Balb/c mice were anesthetized with rumpun (10 mg/kg) and ketamine (100 mg/kg), and 5 × 10^4^ 4T1-Luc tumor cells were injected intravenously. On Days 1, 4, and 7, PBS or 4T1-Luc cancer cell-derived CCNVs or SCCNVs were intradermally injected. On Days 3, 6, and 9, 200 μg of anti-programmed death-ligand 1 (PD-L1) antibodies (BioXCell, NH, USA) was intraperitoneally injected to evaluate the synergistic antitumor efficacy with SCCNVs. To obtain bioluminescence images, prior to imaging, D-luciferin potassium salt (Gold Biotechnology, MO, USA) in sterile water was injected intraperitoneally according to the manufacturer’s protocol^33^. Bioluminescence images were acquired by IVIS spectrum computed tomography (Perkin Elmer), and the total luminescence flux in lung tissues was quantified using Living Image 3.1 software. On Day 15, a 15% India ink solution was injected intratracheally, and the India ink-stained lung tissues were harvested. The harvested lung tissues were washed with distilled water and fixed in Fekete’s solution overnight. Then, images of the India ink-stained lung tissues were obtained, with tumor nodules visualized in white.

### Postsurgery tumor model

Six-week-old C57BL/6 mice were anesthetized with rumpun (10 mg/kg) and ketamine (100 mg/kg) and subcutaneously injected with B16F10 cancer cells (5 × 10^5^ cells in 100 μL PBS per mouse) in the right flank. On Day 14, when the tumor volume reached ~500 mm^3^, the mice were anesthetized with rumpun and ketamine, and the tumor tissues were excised. On Day 17, the mice were randomly divided into three groups, and they were intradermally injected with PBS, CCNVs or SCCNVs (20 μg of nanovesicles in 50 μl of PBS) on Day 17 and Day 23. Tumor size was measured every 2 days using a digital caliper and computed according to the ellipsoidal calculation formula: *V* = 0.5 × (longest diameter) × (shortest diameter). Mouse survival was monitored for 35 days. The mice bearing tumors exceeding 2,500 mm^3^ in size were euthanized with CO_2_ inhalation. For immunohistochemical staining of tumor tissues, tumor tissues were harvested on Day 27. The tumor tissues were analyzed with the DeadEnd™ Fluorometric TUNEL System (Promega, WI, USA) according to the manufacturer’s protocol. For the postsurgery 4T1 breast cancer model, 5 × 10^5^ cells in 100 μL of PBS per mouse were inoculated into the right flank. When the tumor size reached ~200 m^3^, the tumor tissues were excised, and PBS or 4T1 cancer cell-derived CCNVs or SCCNVs were injected. Tumor size was measured every 3 days.

### Statistical analysis

Unless described otherwise, data are presented as the mean ± standard deviation (SD). Data were analyzed through one-way analysis of variance (ANOVA) with Tukey’s multiple comparisons test to calculate *P*-values for comparisons among more than two groups. Two-way ANOVA with Bonferroni’s correction or Holm‒Sidak posttests was used to calculate the *P*-values for comparisons among groups over multiple time points. The log-rank test was used to compare survival differences shown in Kaplan–Meier plots using Prism software (GraphPad, CA, USA).

## Results

**Fig. 2:**
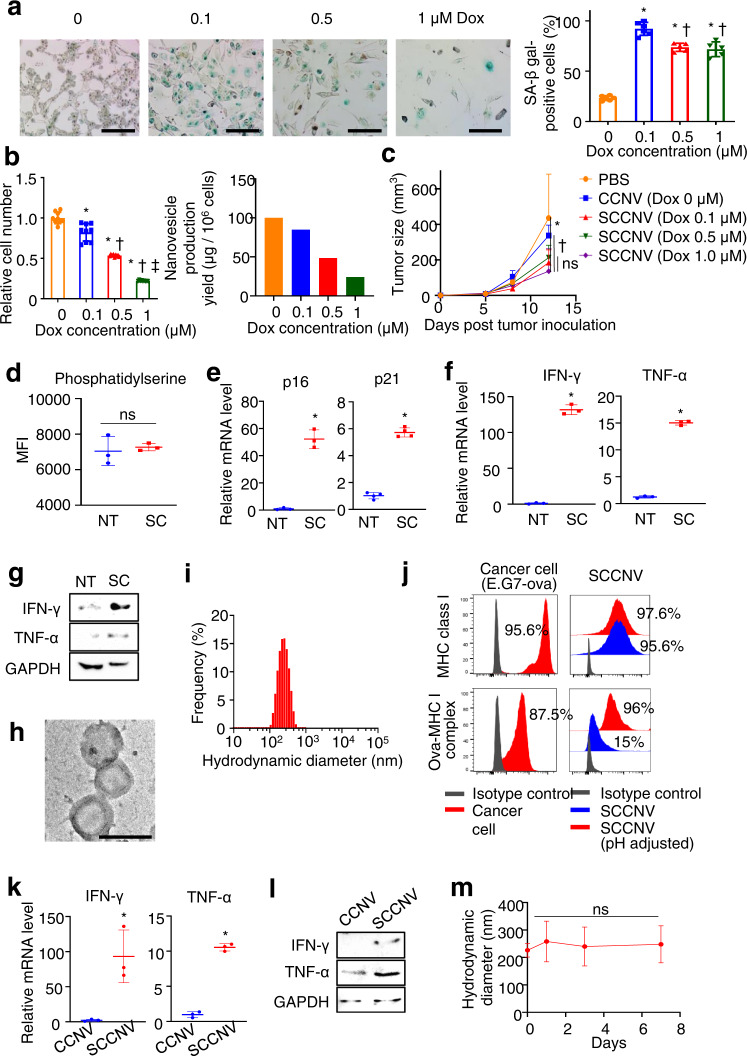
Characterization of SCCNVs. **a** SA β-gal staining (green) of B16F10 cancer cells following senescence induction by treatment with various concentrations of doxorubicin for 4 days. Scale bars = 100 μm. **b** Optimization of the Dox concentration: the cell number relative to the initial cell number and nanovesicle production yield of B16F10 cancer cells after doxorubicin treatment at various concentrations for 4 days. **c** Optimization of the Dox concentration: antitumor efficacy of SCCNVs produced from cancer cells treated with various doxorubicin concentrations. *n* = 3–5. **d** Phosphatidylserine exposure on the B16F10 cancer cell surface after senescence induction with 0.1 μM doxorubicin, as evaluated by flow cytometric analysis. NT no treatment, SC senescence induction. *n* = 3. mRNA levels of (**e**) p16 and p21, (**f**) IFN-γ and TNF-α and (**g**) protein levels of IFN-γ and TNF-α in B16F10 cancer cells after senescence induction with 0.1 μM doxorubicin. NT no treatment, SC senescence induction. **h** Transmission electron microscopy image of SCCNVs. Scale bar = 250 nm. **i** Hydrodynamic diameter of SCCNVs. **j** Detection of the ovalbumin epitope presented on MHC class I expressed by E.G7-OVA cancer cells by FACS analysis. Following extrusion through nanosized pores, 15% of SCCNVs were positive for the OVA-MHC I complex. pH adjustment enabled the detached OVA in the supernatant to be reloaded on MHC I molecules, increasing the proportion of OVA-MHC I-positive SCCNVs to 96%. Relative (**k**) mRNA and (**l**) protein levels of IFN-γ and TNF-α in CCNVs and SCCNVs. **m** Hydrodynamic diameter of SCCNVs incubated in 10% (v/v) serum-containing buffer for various time periods, showing the colloidal stability of the SCCNVs. ns not significant. Data represent the mean ± SD. Statistical significance was calculated by Student’s *t*-tests (**e**, **f**, and **k**) or one-way analysis of variance (ANOVA) with Tukey’s multiple comparisons test (**a**, **b**, and **m**). *P < 0.05 versus NT or 0 μM doxorubicin in (**a**–**f**), versus CCNVs in **i**. †*P* < 0.05 versus 0.1 μM in **a** and **b**, ‡*P* < 0.05 versus 0.5 μM in **b**.

To induce senescence in B16F10 melanoma cells, cells were treated with various concentrations of doxorubicin. Senescence-associated beta-galactosidase (SA-β-gal) staining showed that doxorubicin concentrations higher than 0.1 μM successfully induced senescence. A doxorubicin concentration of 0.1 μM resulted in the highest cell viability and nanovesicle production yield (Fig. 2a, b). The in vivo antitumor efficacy of SCCNVs was not significantly different across the 0.1~1.0 μM range of doxorubicin concentrations used for SCCNV preparation (Fig. 2c). Thus, we chose 0.1 μM as the optimal concentration of doxorubicin and used this concentration in subsequent experiments. This concentration was 100 times lower than that used for clinical chemotherapy, and the residual doxorubicin after doxorubicin treatment of cells was removed prior to the use of SCCNVs in experiments. Therefore, doxorubicin itself would not exert unexpected antitumor effects in SCCNV therapy. Exposure of phosphatidylserine on the B16F10 cell surface did not show a significant change before and after 0.1 μM doxorubicin treatment (Fig. 2d), indicating that 0.1 μM doxorubicin treatment did not induce severe apoptosis of B16F10 cells. The mRNA expression levels of p16 and p21, cellular senescence markers, were increased in B16F10 cells treated with 0.1 μM doxorubicin (Fig. 2e). In addition, the mRNA and protein levels of IFN-γ and TNF-α, both of which are SASP markers induced in senescent cells, were increased in B16F10 cells treated with 0.1 μM doxorubicin (Fig. 2f, g). Transmission electron microscopy (TEM) analysis showed a unilamellar structure with lipid bilayers of SCCNVs (Fig. 2h; Supplementary Fig. 1). SCCNVs showed hydrodynamic diameters of 226.1 ± 24.6 nm, as revealed by dynamic light scattering analysis (Fig. 2i), and the zeta potential of SCCNVs was -20.2 ± 8.7 (Supplementary Fig. 2). Coomassie blue staining revealed that senescence induction did not change the protein profiles of B16F10 cancer cells, and SCCNVs preserved most of the proteins from senescent B16F10 cancer cells, with a small variation possibly due to the relative increase in cell-surface proteins through nanovesicle production (Supplementary Fig. 3)^23^. Next, we investigated whether the antigens presented by MHC class I on cancer cells were preserved on SCCNVs by FACS analysis (Fig. 2j). SCCNVs were produced from E.G7-OVA cancer cells, which present the ovalbumin (OVA) epitope (SIINFEKL) on MHC class I molecules. Extrusion through nanosized pores induced antigen detachment from MHC class I and reduced the portion of OVA–MHC class I complex-positive SCCNVs to 15%. Following extrusion, adjustment of the pH in the SCCNV-containing solution to 5.5 for 30 min^31,32^ induced complete reloading of the antigens on MHC class I. There was no significant difference in antitumor efficacy between SCCNVs and pH-adjusted SCCNVs, indicating that the partial loss of antigens presented on MHC class I did not affect the antitumor efficacy of SCCNVs (Supplementary Fig. 4). The increased IFN-γ and TNF-α levels in senescence-induced cells were preserved in SCCNVs at both the mRNA and protein levels (Fig. 2k, l). The amounts of IFN-γ and TNF-α in SCCNVs were quantified by enzyme-linked immunosorbent assay (ELISA), and approximately 4.56 pg of IFN-γ and 0.14 pg of TNF-α were included in 1 µg of SCCNVs (Supplementary Fig. 5). Finally, the hydrodynamic diameter of SCCNVs was monitored for one week, and the results showed that the size of SCCNVs was stably maintained in serum-containing buffer for at least 7 days (Fig. 2m).

**Fig. 3:**
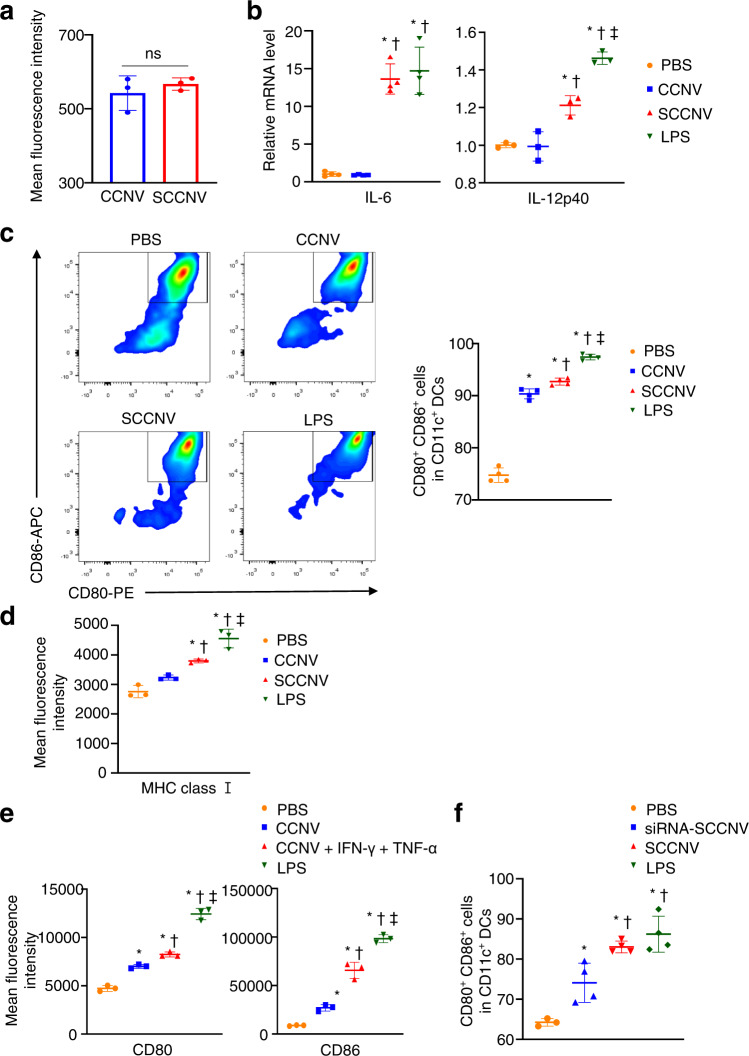
Effective DC maturation in vitro by SCCNVs. **a** In vitro uptake of CCNVs and SCCNVs by DCs for 10 min, as evaluated by flow cytometric analysis. *n* = 3. **b** mRNA levels of DC maturation markers (IL-6 and IL-12p40) in BMDCs treated with PBS, CCNV, SCCNV, or LPS for 4 h in vitro, as evaluated by qRT‒PCR. *n* = 3-4. **c** Representative flow cytometry plot and percentage of CD80 and CD86 double-positive cells in BMDCs treated with PBS, CCNV, SCCNV, or LPS for 24 h in vitro, evaluated by flow cytometric analysis. *n* = 4. **d** Flow cytometric analysis of MHC class I expression of BMDCs treated with PBS, CCNVs, SCCNVs, or LPS for 24 h in vitro, as evaluated by flow cytometric analysis. *n* = 3. **e** Flow cytometric analysis showing that the enhanced DC maturation induced by SCCNVs is likely due to IFN-γ and TNF-α contained in SCCNVs. Exogenous IFN-γ and TNF-α were added to the CCNV + IFN-γ + TNF-α group. *n* = 4. **f** Effects of various treatments for 24 h on DC maturation, as evaluated by flow cytometric analysis. siRNA-SCCNVs are nanovesicles derived from senescent cancer cells transfected with siRNA specific for IFN-γ and TNF-α. *n* = 4. In (**b**–**f**), LPS was used as the positive control. Data represent the mean ± SD. Statistical significance was calculated by Student’s *t*-tests or one-way ANOVA with Tukey’s multiple comparisons test. *P < 0.05 versus PBS; †*P* < 0.05 versus CCNVs; ‡*P* < 0.05 versus SCCNVs in **b**, **c**, **d** and **f** and versus CCNVs + IFN-γ, TNF-α in **e**. ns not significant.

Next, we investigated whether SCCNVs can promote DC maturation in vitro. DCs can internalize both SCCNVs and cancer cell-derived nanovesicles (CCNVs), and the uptake efficiency was not different between SCCNVs and CCNVs (Fig. 3a). SCCNV-treated DCs showed significantly higher mRNA levels of IL-6 and IL-12p40 (DC maturation markers) than CCNV-treated DCs, indicating that the SCCNVs stimulated DC maturation (Fig. 3b). CCNVs did not stimulate sufficient DC maturation since there were no differences in the mRNA levels of IL-6 and IL-12p40 between the PBS and CCNV groups. FACS analysis of CD86, CD80 and MHC class I expressed on DCs confirmed the stimulation of DC maturation by SCCNVs (Fig. 3c, d). FACS analysis revealed that TNF-α and IFN-γ significantly enhanced DC maturation, as shown comparing the CCNV group and the CCNV + TNF-α + IFN-γ group (Fig. 3e). In addition, siRNA-SCCNVs were produced from B16F10 cancer cells that were treated with doxorubicin and transfected with siRNAs specific for IFN-γ and TNF-α. siRNA transfection induced knockdown of IFN-γ and TNF-α in doxorubicin-treated B16F10 cancer cells (Supplementary Fig. 6). siRNA-SCCNVs were less effective in inducing DC maturation than SCCNVs (Fig. 3f). These data indicate that the enhanced DC maturation induced by SCCNV treatment is due to IFN-γ and TNF-α contained in the SCCNVs. This suggests that a significant portion of the stimulatory effect of SCCNVs on DC maturation is due to TNF-α and IFN-γ delivered by the SCCNVs.

**Fig. 4:**
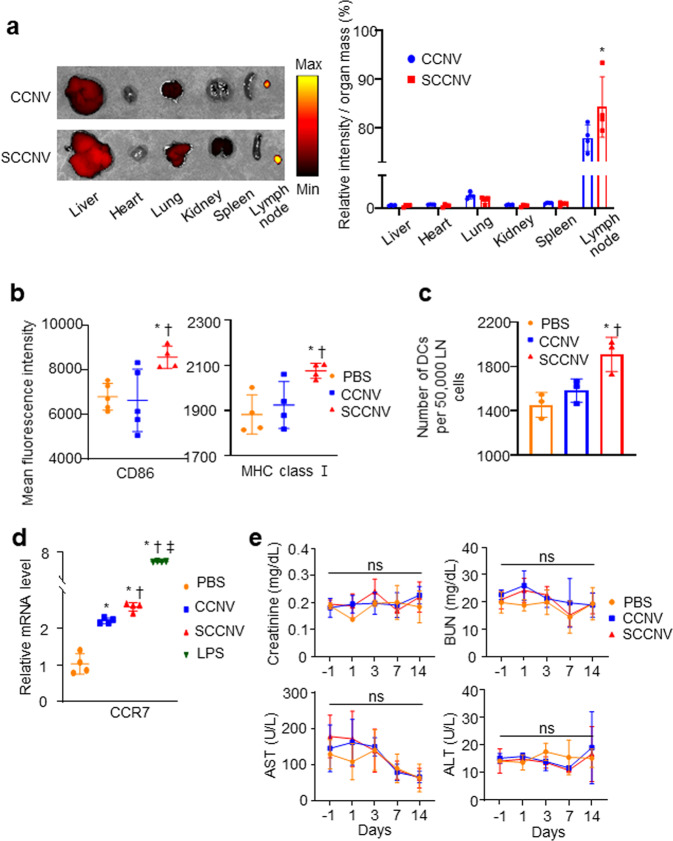
Effective homing of SCCNVs to lymph nodes, DC maturation in vivo, and SCCNV toxicity. **a** Ex vivo imaging at 24 h after intradermal injection of fluorescently labeled CCNVs and SCCNVs. Relative fluorescence was divided by organ mass. *n* = 4. **b** Maturation marker expression in dendritic cells in the draining lymph nodes 3 days after intradermal injection of PBS, CCNVs, or SCCNVs, as evaluated by flow cytometric analysis. *n* = 4–5. **c** Number of dendritic cells in the draining lymph nodes 7 days after intradermal injection of PBS, CCNVs, or SCCNVs, as evaluated by flow cytometric analysis. *n* = 3. **d** mRNA level of CCR7, a representative marker of DC migration to secondary lymphoid organs, in BMDCs treated with PBS, CCNV, SCCNV, or LPS for 24 h in vitro. *n* = 4. **e** Serum levels of creatinine, BUN, AST, and ALT in mice treated with intradermal injection of PBS, CCNVs, or SCCNVs three times. *n* = 4. Statistical significance was calculated by one-way analysis of variance (ANOVA) with Tukey’s multiple comparisons test or two-way analysis of variance with Bonferroni posttests. *P < 0.05 versus CCNVs in **a** and versus PBS in (**b**–**d**); †*P* < 0.05 versus CCNVs in (**b**–**d**); ‡*P* < 0.05 versus SCCNVs in **d**. ns not significant.

Ex vivo imaging performed 24 h after intradermal injection of fluorescently labeled SCCNVs or CCNVs showed that SCCNV migration to the draining lymph nodes was significantly higher than that of CCNVs (Fig. 4a). Several studies have shown that nanoparticles larger than 100 nm migrate into the lymph nodes by cellular uptake^38–40^. Therefore, this result may be attributed to the fact that SCCNV uptake by immature DCs stimulates DC maturation, leading to the subsequent migration of mature DCs to the draining lymph nodes, while CCNV uptake does not stimulate DC maturation. To investigate the in vivo maturation of DCs, DCs in the draining lymph nodes were analyzed 3 days after intradermal injection of SCCNVs. DCs harvested from SCCNV-injected mice showed relatively high expression of CD86 and MHC class I (Fig. 4b), revealing that SCCNVs facilitated DC maturation in vivo. Seven days after intradermal injection, the DC population in the draining lymph nodes was significantly larger in the SCCNV injection group than in the CCNV or PBS injection group (Fig. 4c). The mRNA level of CCR7, a marker related to DC migration to secondary lymphoid organs, was significantly increased in the SCCNV injection group compared to the CCNV or PBS injection group (Fig. 4d). Injected SCCNVs did not induce toxicity in the liver, kidneys or other major organs (Fig. 4e; Supplementary Fig. 7).

**Fig. 5:**
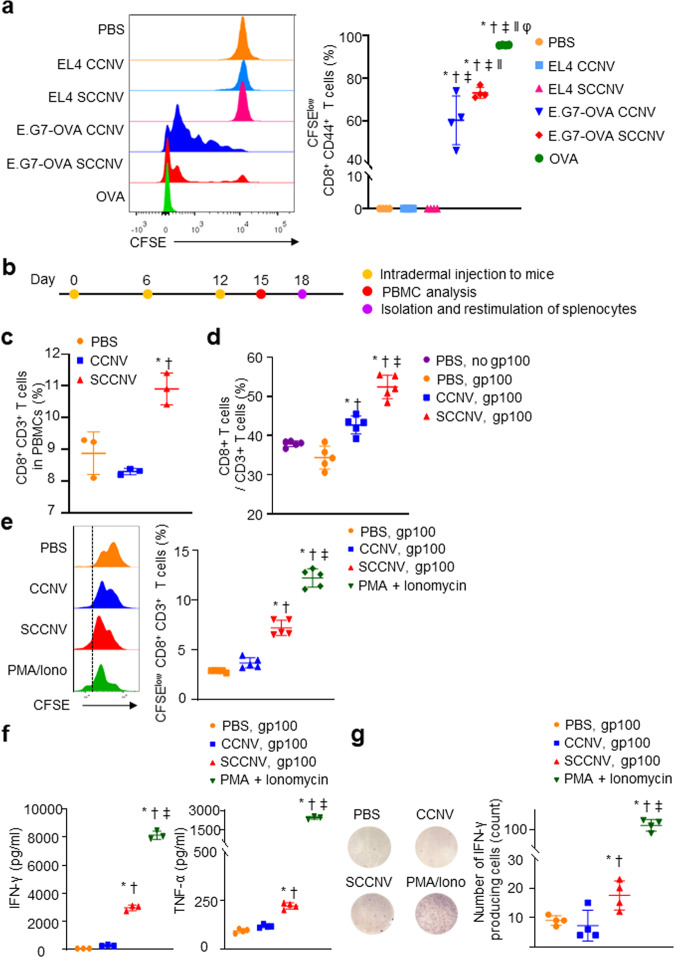
Effective activation of tumor-specific CD8^+^ T cells by SCCNVs in vitro and in vivo. **a** Flow cytometric analysis of the in vitro proliferation of naïve OT-I CD8 + T cells labeled with CFSE and subsequently cocultured for 72 h with splenic DCs that had been pulsed with PBS, ovalbumin peptide (OVA), CCNVs or SCCNVs. CCNVs and SCCNVs were produced from EL4-cancer cells or E.G7-OVA cancer cells. OVA was used as the positive control. **b** Timeline for the C57BL/6 mouse immunization study. **c** Flow cytometric analysis of CD8^+^ T cells in the PBMCs of mice immunized with intradermal injection of PBS, B16F10 cancer cell-derived CCNVs, or SCCNVs. **d** Ratio of CD8^+^/CD3^+^ T cells in splenocytes harvested from mice that were immunized with PBS, B16F10 cancer cell-derived CCNVs, or SCCNVs and subsequently restimulated in vitro with the gp100 peptide (the tumor antigen of B16F10 cancer cells) for 72 h. **e** In vitro proliferation of CD8^+^ T cells during the restimulation of CFSE-labeled splenocytes harvested from mice that had been immunized with PBS, B16F10 cancer cell-derived CCNVs, or SCCNVs. PMA/ionomycin (T-cell activation-inducing agents) served as the positive control. **f** Levels of IFN-γ and TNF-α, both of which are CD8^+^ T-cell activation markers, in the culture medium following the in vitro restimulation of splenocytes. The levels of IFN-γ and TNF-α were evaluated with ELISA. **g** ELISpot analysis of splenocytes harvested from mice immunized with various agents. The number of IFN-γ-producing cells per well was evaluated (*n* = 4). Statistical significance was calculated by one-way ANOVA with Tukey’s multiple comparisons test. *P < 0.05 versus PBS in (**a**–**c**) and (**e**–**g**) or versus PBS, no pg100 in **d**; †*P* < 0.05 versus EL4 cancer cell-derived CCNVs in **a**, versus CCNV in **c**, **e**, **f** and **g**, and versus PBS, gp100 in **d**; ‡*P* < 0.05 versus EL4 cancer cell-derived SCCNVs in (**a**), versus CCNVs in (**d**), and versus SCCNV in **e**, **f** and **g**; ‖*P* < 0.05 versus CCNVs in **a**; φ*P* < 0.05 versus SCCNVs in **a**.

Given that SCCNVs stimulated DC maturation in vivo, we next investigated whether SCCNVs could promote the activation of tumor-specific T cells. Carboxyfluorescein succinimidyl ester (CFSE)-labeled OT-1 CD8 + T cells were cocultured with splenic DCs that had been pulsed with PBS, OVA peptide, CCNVs or SCCNVs. The CCNVs and SCCNVs were derived from EL4-cancer cells or E.G7-OVA cancer cells. The group containing SCCNV-pulsed DCs showed significantly higher activation of OVA-specific T cells (OT-1 transgenic mouse-derived cells) than the other groups, as shown by the lower mean fluorescence intensity of CFSE-stained CD8^+^ T cells in the SCCNV group (Fig. 5a). The positive control (the OVA peptide group) confirmed that T-cell proliferation was due to OVA presented by DCs. To investigate whether SCCNVs promote T-cell activation in vivo, B16F10 cancer cell-derived SCCNVs, B16F10 cancer cell-derived CCNVs, or PBS was injected intradermally into mice, and peripheral blood mononuclear cells (PBMCs) and splenocytes were collected and analyzed (Fig. 5b). After immunization, the population of CD8^+^ T cells in PBMCs was significantly increased in the SCCNV injection group (Fig. 5c). Splenocytes isolated from immunized mice were restimulated in vitro with gp100 peptides (tumor antigen of B16F10 cancer cells) or PMA/ionomycin as a positive control. The SCCNV injection group showed a higher ratio of CD8^+^ T cells/CD3 + T cells and stronger proliferation of CD8^+^ T cells (Fig. 5d, e). Enzyme-linked immunosorbent assay (ELISA) analysis of supernatants from cultures of gp100-pulsed splenocytes isolated from various groups of mice revealed higher IFN-γ and TNF-α levels in SCCNV-immunized mice (Fig. 5f), and an enzyme-linked immunospot (ELISpot) assay showed a higher number of IFN-γ-producing gp100-specific T cells in the spleen of SCCNV-immunized mice than in that of CCNV-immunized mice (Fig. 5g). Together, these results indicated that SCCNV immunization more effectively stimulated the activation of vaccine antigen (gp100)-specific CD8^+^ T cells than did CCNV immunization.

**Fig. 6:**
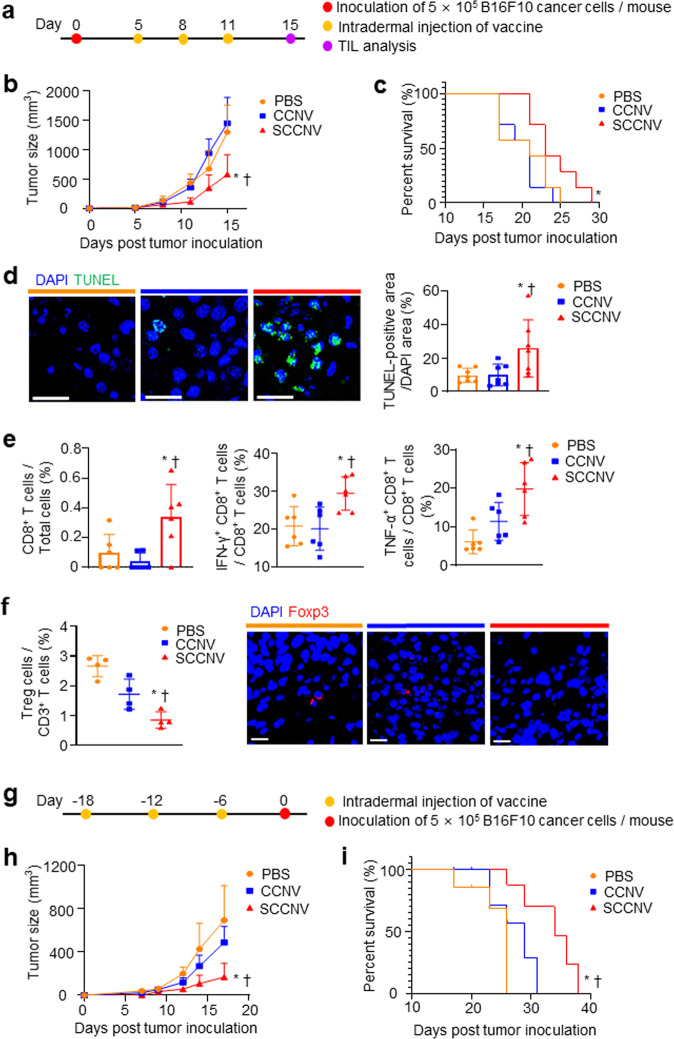
Therapeutic efficacy of SCCNVs in a therapeutic melanoma murine model and a prophylactic melanoma murine model. **a** Therapeutic tumor modeling and vaccination timeline for (**b**–**f**). **b** Tumor growth profile. *n* = 6. **c** Animal survival rate. *n* = 6. **d** TUNEL staining of tumor tissues harvested on Day 15. *n* = 7. Scale bars = 100 μm. **e** Immunohistochemical staining for CD8 + T cells in tumor tissues and flow cytometric TIL analysis of tumor tissues harvested on Day 15. *n* = 6. **f** Percentage of Treg cells measured in the flow cytometric TIL analysis (*n* = 4) and immunohistochemical staining for Foxp3 in tumor tissues harvested on Day 15. Scale bars = 50 μm. **g** Vaccination and prophylactic tumor modeling timeline for (**h**) and (**i**). **h** Tumor growth profile. *n* = 5. **i** Animal survival rate. *n* = 5. Statistical significance was calculated by the log-rank (Mantel–Cox) test (c and i), one-way ANOVA with Tukey’s multiple comparisons test (**d**–**f**) or by two-way ANOVA with Bonferroni posttests (**b** and **h**). *P < 0.05 versus PBS; †*P* < 0.05 versus CCNVs.

Next, we investigated whether SCCNVs can suppress tumor growth in therapeutic and prophylactic melanoma mouse models. SCCNVs were injected intradermally into B16F10 tumor-bearing mice (Fig. 6a). SCCNVs significantly suppressed tumor growth in vivo and improved animal survival (Fig. 6b, c; Supplementary Fig. 8). A terminal deoxynucleotidyl transferase dUTP nick end labeling (TUNEL) staining assay showed a significantly higher density of apoptotic cells in tumor tissues in the SCCNV-immunized group (Fig. 6d). Immunohistochemical (IHC) staining and tumor-infiltrating lymphocyte (TIL) analysis of tumor tissues showed significantly higher densities of activated (IFN-γ- or TNF-α-positive) cytotoxic T cells in the SCCNV-immunized group (Fig. 6e; Supplementary Fig. 9 and 10). Moreover, TIL analysis and IHC staining showed that the regulatory T-cell density was significantly lower in the SCCNV-immunized group, indicating a proinflammatory microenvironment in the SCCNV-immunized group (Fig. 6f). Furthermore, in the prophylactic model, mice that underwent immunization were challenged with B16F10 melanoma cells (Fig. 6g). SCCNV immunization inhibited tumor growth more effectively and resulted in a significantly higher survival rate than CCNV or PBS immunization (Fig. 6h, i; Supplementary Fig. 11).

**Fig. 7:**
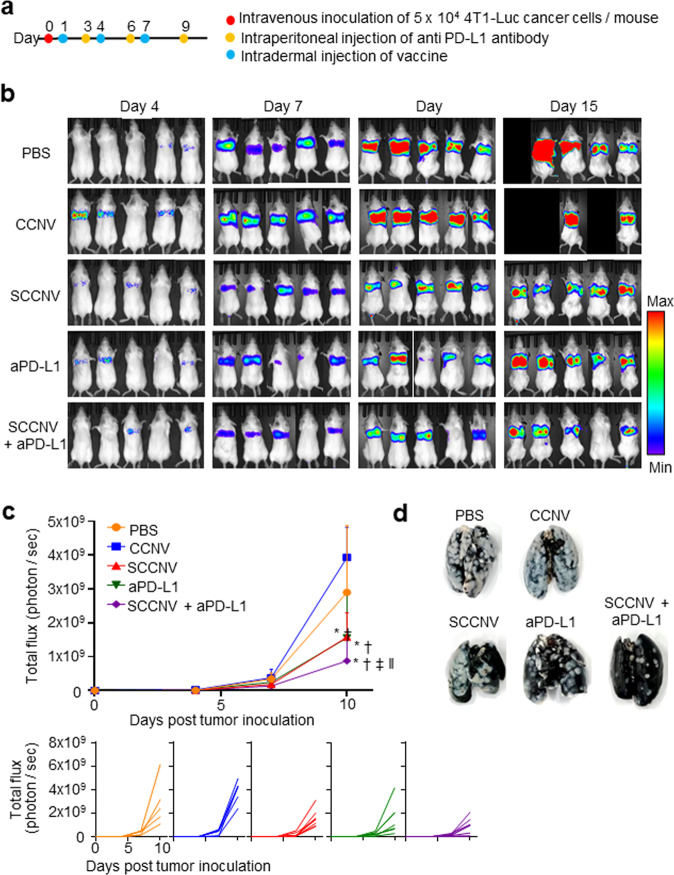
Inhibition of tumor metastasis by SCCNV injection in murine models. **a** Timeline for metastasis modeling of 4T1-Luc tumors with vaccination.**b** Representative bioluminescence images and **c** luminescence flux showing tumor metastasis to the lungs in 4T1-Luc tumor cell-inoculated mice at various time points. *n* = 5–6. **d** Images of lungs harvested on Day 15 and stained with India ink. Tumor nodules were stained white, and normal lung tissues were stained black. Statistical significance was calculated by two-way ANOVA with Holm‒Sidak posttests. *P < 0.05 versus PBS; †*P* < 0.05 versus CCNVs; ‡*P* < 0.05 versus SCCNVs; ‖*P* < 0.05 versus aPD-L1.

Next, we investigated whether SCCNV injection could suppress the metastatic growth of 4T1-Luc breast cancer cells, and anti-programmed death-ligand 1 (PD-L1) antibodies were administered to assess combination therapy (Fig. 7a). PD-L1 expression on 4T1-Luc cancer cells was confirmed by FACS analysis (Supplementary Fig. 12). Live bioluminescence imaging of metastatic tumor-bearing mice revealed that SCCNV vaccination significantly suppressed lung metastasis (Fig. 7b, c). Lung metastasis visualization with India ink staining confirmed the live bioluminescence imaging results (Fig. 7d). Importantly, SCCNV monotherapy produced a therapeutic outcome similar to that achieved with anti-programmed death-ligand 1 (PD-L1) antibody therapy at a clinical dose in this metastatic model. SCCNV vaccination successfully synergized with the anti-PD-L1 antibody, which prevented inactivation of tumor-reactive T cells in the immunosuppressive tumor microenvironment (Fig. 7b-d).

**Fig. 8:**
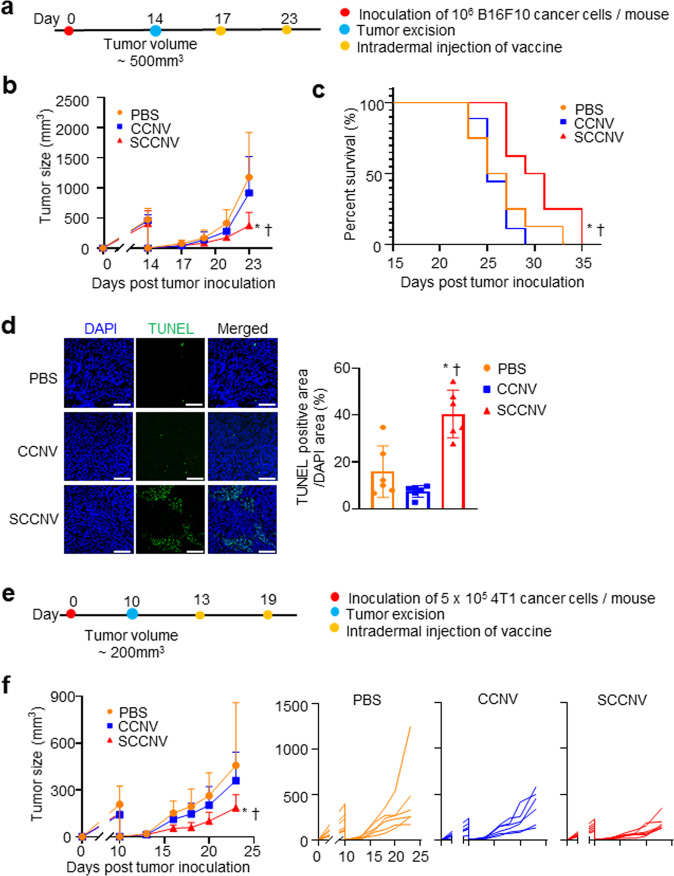
Inhibition of postsurgery recurrence of melanoma and 4T1 tumors by SCCNV injection in murine models. **a** Timeline of postsurgery recurrence in the B16F10 tumor model for (**b**–**d**). **b** Tumor growth profile. *n* = 13. **c** Animal survival rate. *n* = 8. **d** TUNEL staining of tumor tissues harvested on Day 27. *n* = 6. Scale bars = 100 μm. **e** Timeline of postsurgery recurrence in the 4T1 tumor model. **f** Tumor growth profile. *n* = 6. Statistical significance was calculated by two-way ANOVA with Bonferroni posttests (**b** and **f**), the log-rank (Mantel–Cox) test (**c**), or one-way ANOVA with Tukey’s multiple comparisons test (**d**). *P < 0.05 versus PBS; †*P* < 0.05 versus CCNVs.

Finally, we investigated whether SCCNV vaccination could inhibit tumor recurrence in postsurgery models of B16F10 melanoma and 4T1-Luc breast cancer. When the melanoma tumor volume reached ~500 mm^3^, the tumor tissues were removed, and the mice were vaccinated with B16F10 cell-derived SCCNVs (Fig. 8a). SCCNV vaccination inhibited tumor recurrence more effectively (Fig. 8b; Supplementary Fig. 13) and produced significantly higher survival rates (Fig. 8c) than PBS or CCNV injection. A TUNEL staining assay showed that SCCNV injection resulted in a significantly higher density of apoptotic cells in tumor tissues (Fig. 8d). Additionally, in the 4T1-Luc breast cancer model, SCCNV vaccination inhibited tumor recurrence more effectively than PBS or CCNV injection (Fig. 8e, f).

## Discussion

As exosomes secreted by cancer cells in the TME may stimulate tumor growth by mediating crosstalk between cancer cells and immune cells in the TME^41^, and SCCNVs may be similar to cancer cell-derived exosomes^42^. However, SCCNVs did not stimulate tumor growth because SCCNVs were injected intradermally and internalized by DCs in a tumor-free region, which prevents crosstalk between SCCNVs and immune cells in the TME.

Tumor lysates, which may have a similar composition to cancer cell-derived nanovesicles, can also be used as a potential TCV to deliver tumor antigens. However, tumor lysate vaccination generally results in low-efficiency DC activation and, in turn, weak antitumor immunity^43^. In contrast, SCCNVs can deliver tumor antigens and proinflammatory cytokines (IFN-γ and TNF-α) simultaneously to DCs, leading to a higher efficiency of DC activation. Effective DC activation led to effective activation of tumor antigen-specific T cells and tumor inhibition in various mouse tumor models, including a prophylactic model, primary tumor model, metastasis model and postsurgery tumor recurrence model. These results show the potential of SCCNVs as a TCV.

SCCNVs may be clinically feasible for development into a personalized TCV. In the present study, we treated tumor-bearing model mice with SCCNVs derived from in vitro senescence-induced cancer cell lines because most of the antigens of these immortalized cell lines are preserved over a short period^44^. The heterogeneity of tumor cells and the difficulty of neoantigen identification make tumor treatment difficult. However, SCCNVs obtained from autologous tumor tissues would provide a patient-specific spectrum of tumor antigens with no need for neoantigen identification. Therefore, SCCNVs may be applied in the context of liquid tumors, such as acute myeloid leukemia, which have a small chance of being treated with immunotherapy due to the limited identification of tumor antigens and low response rate for immune checkpoint blockade^45,46^. For solid tumors, SCCNVs, as personalized TCVs, could be prepared from autologous tumor tissues removed during surgery in end-stage cancer patients in whom surgical removal of tumor tissues may be necessary. In addition, SCCNVs could be used to eliminate metastatic cancer cells and prevent tumor recurrence in end-stage cancer patients, in whom metastasis or tumor recurrence is often observed.

Currently, several ongoing clinical trials are evaluating the efficacy of TCVs with neoantigens in the form of peptides (NCT03639714, NCT03223103 and NCT02721043), mRNA (NCT04163094) or DNA (NCT04015700 and NCT04251117). However, the development of TCVs employing neoantigens is limited by the difficulty and labor intensiveness of this approach, as well as patient-to-patient variations in neoantigens. Only a small fraction (~1–2%) of mutations in cancer cells induce antitumor immune responses^15^, and only a small number of neoantigens are shared among cancer patients^10^. TCV therapy employing a single neoantigen may fail for tumors that undergo constant mutation and consequently evade recognition by vaccine-activated T cells^47^. In contrast, SCCNVs produced from autologous cancer cells can avoid the labor-intensive and time-consuming processes needed for the identification of immunogenic neoantigens in individual patients and be free from the concern of patient-to-patient variations.

In conclusion, this study suggests the use of SCCNVs as a potential personalized TCV strategy. SCCNVs prepared from autologous (possibly heterogeneous) cancer cells can deliver a broad spectrum of patient-specific neoantigens and safe adjuvants and effectively activate tumor-reactive T cells. SCCNVs may show clinical benefit since SCCNVs showed an efficacy similar to that of immune checkpoint blockade therapy (anti-PD-L1 antibody) in our animal study (Fig. 7). Moreover, immune checkpoint inhibitors that prevent T-cell exhaustion in the immunosuppressive TME can function synergistically with tumor-reactive T cells activated by SCCNVs to inhibit tumor growth (Fig. 7). Therefore, we suggest SCCNVs as a novel personalized cancer vaccine with facile and simple procedures.

## Supplementary information

Supplementary information
