# Supplementary material for: Senescent cancer cell-derived nanovesicle as a personalized therapeutic cancer vaccine
Source: Exp Mol Med. 2023 Mar 1;55(3):541–54. doi: 10.1038/s12276-023-00951-z (PMC10073290; doi:10.1038/s12276-023-00951-z)
Supplement: Supplementary file 1 — Supplementary information [file 12276_2023_951_MOESM1_ESM.pdf]

Supplementary Information for

# Senescent cancer cell-derived nanovesicle as a personalized therapeutic cancer vaccine

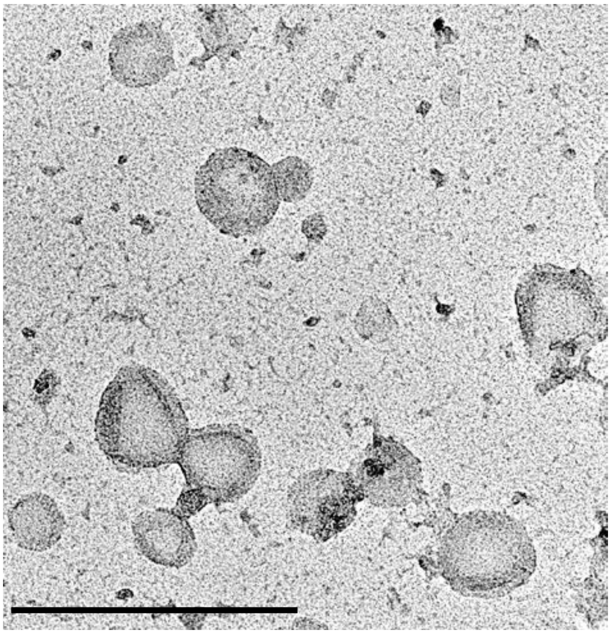

**Supplementary Fig. 1.** TEM image showing multiple SCCNVs. Scale bar = 500 nm.

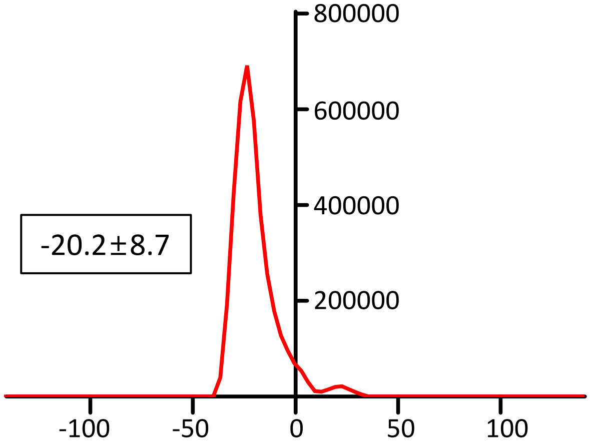


**Supplementary Fig. 2.** Zeta potential analysis of SCCNVs.

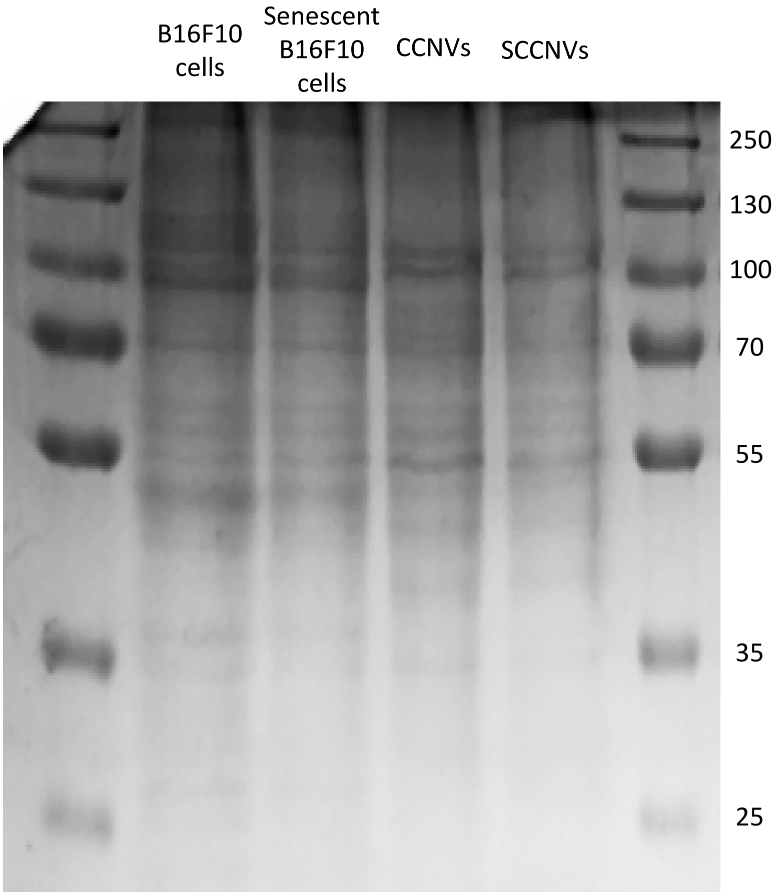


**Supplementary Fig. 3.** Coomassie blue staining of lysates of B16F10 cancer cells, senescence-induced B16F10 cancer cells, CCNVs and SCCNVs. The protein profiles of the lysates show that proteins are preserved in senescent induction step and extrusion step. Differences in some proteins are possibly exosome markers, which are upregulated in nanovesicles.

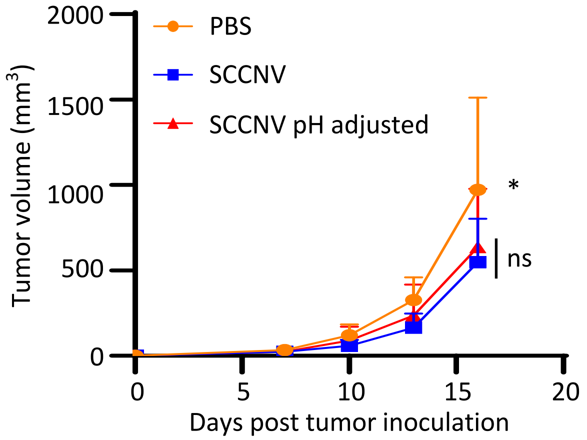


**Supplementary Fig. 4.** In vivo antitumor efficacy of SCCNVs and pH-adjusted SCCNVs. n = 6. * $P < 0.05$ versus SCCNV or SCCNV pH adjusted. ns = Not significant.

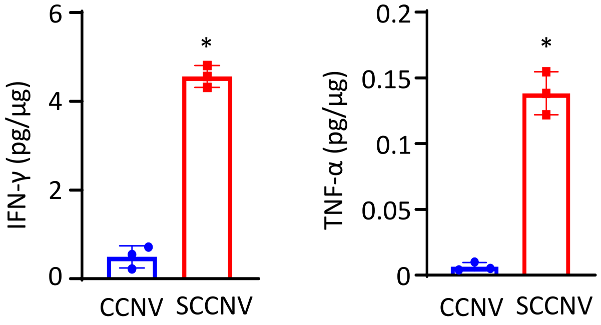


**Supplementary Fig. 5.** Detection of IFN-γ and TNF-α in lysates of CCNV and SCCNV by ELISA. 1μg of SCCNVs contain approximately 4.56 pg of IFN-γ and 0.14 pg of TNF-α. n = 3. Statistical significance was calculated by Student's t-tests.* *P* < 0.05 versus CCNV.

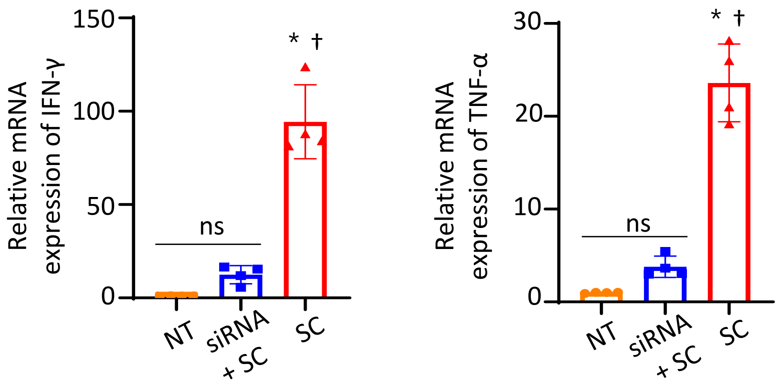


**Supplementary Fig. 6.** Effect of knock-down of IFN-γ and TNF-α by siRNA transfection on the doxorubicin-mediated senescence induction of B16F10 cancer cells, as evaluated by qRT-PCR (n = 4). Statistical significance was calculated by one-way analysis of variance (ANOVA) with Tukey's significant difference multiple comparisons. * $P < 0.05$ versus NT, † P<0.05 versus siRNA + SC. ns : no significance.

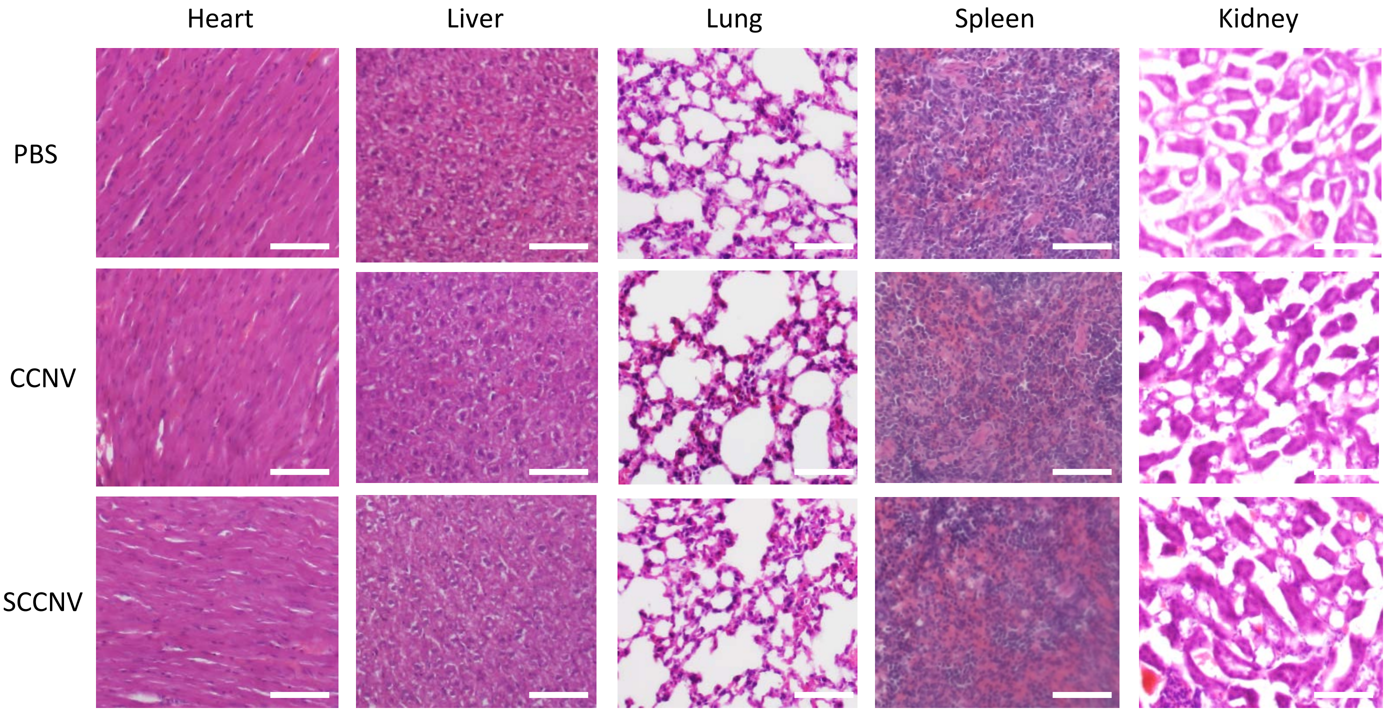


**Supplementary Fig. 7.** *In vivo* toxicity of intradermally injected CCNV and SCCNV. H&E staining of major organs at day 14. Scale bars: 100 µm.

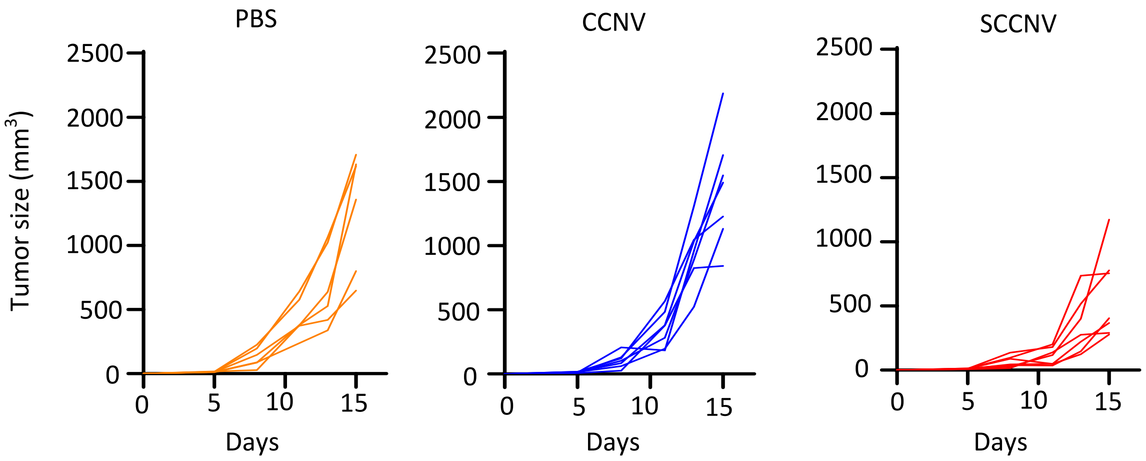


**Supplementary Fig. 8.** Individual tumor growth profiles of B16F10 therapeutic model shown in Figure 6B. n = 6.

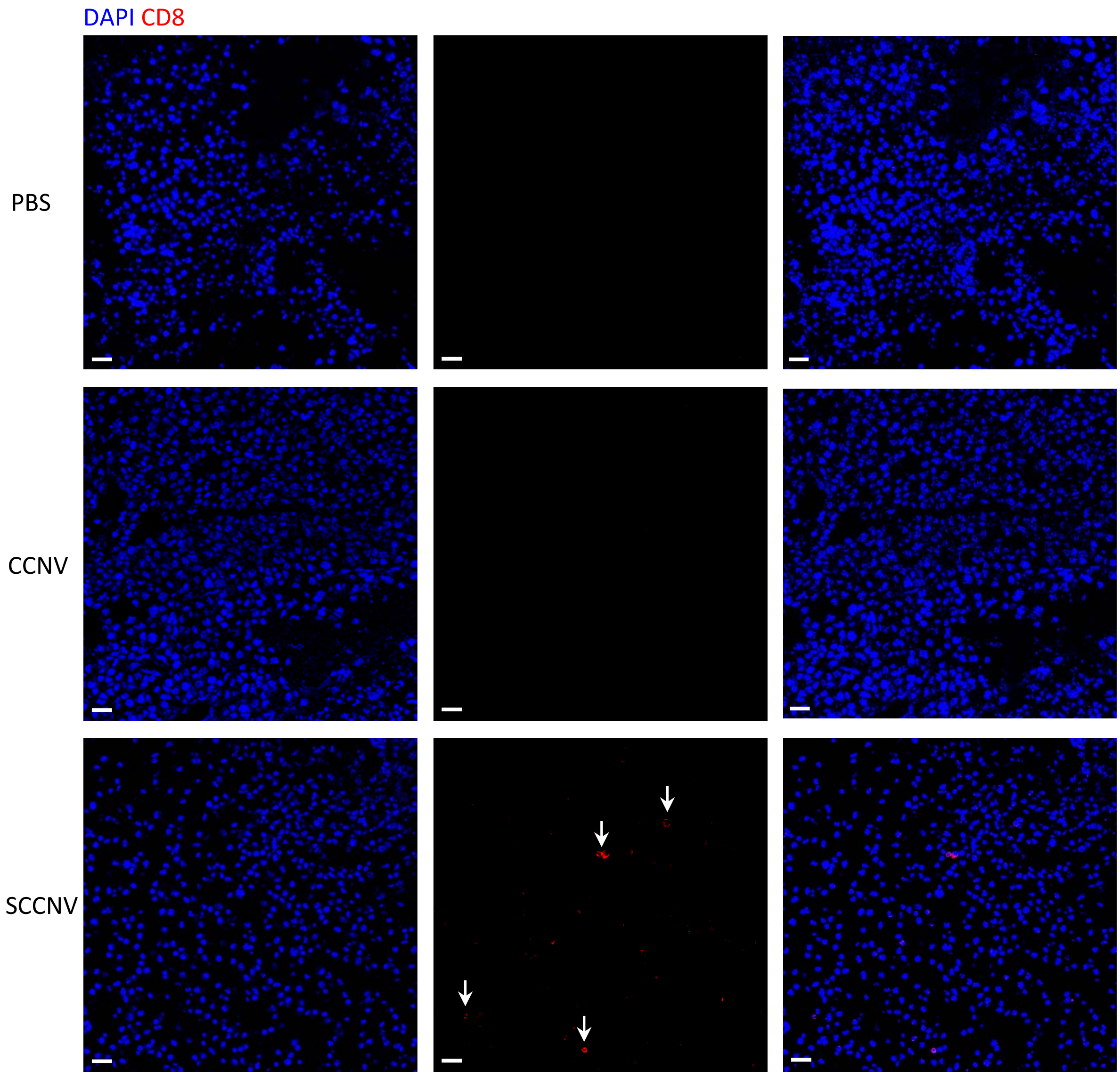


**Supplementary Fig. 9.** Immunohistochemistry for CD8$^{+}$ T cells in B16F10 tumor tissues retrieved on day 15 shown in Figure 6E . Arrows indicate CD8+ cells. Scale bars = 100 μm.

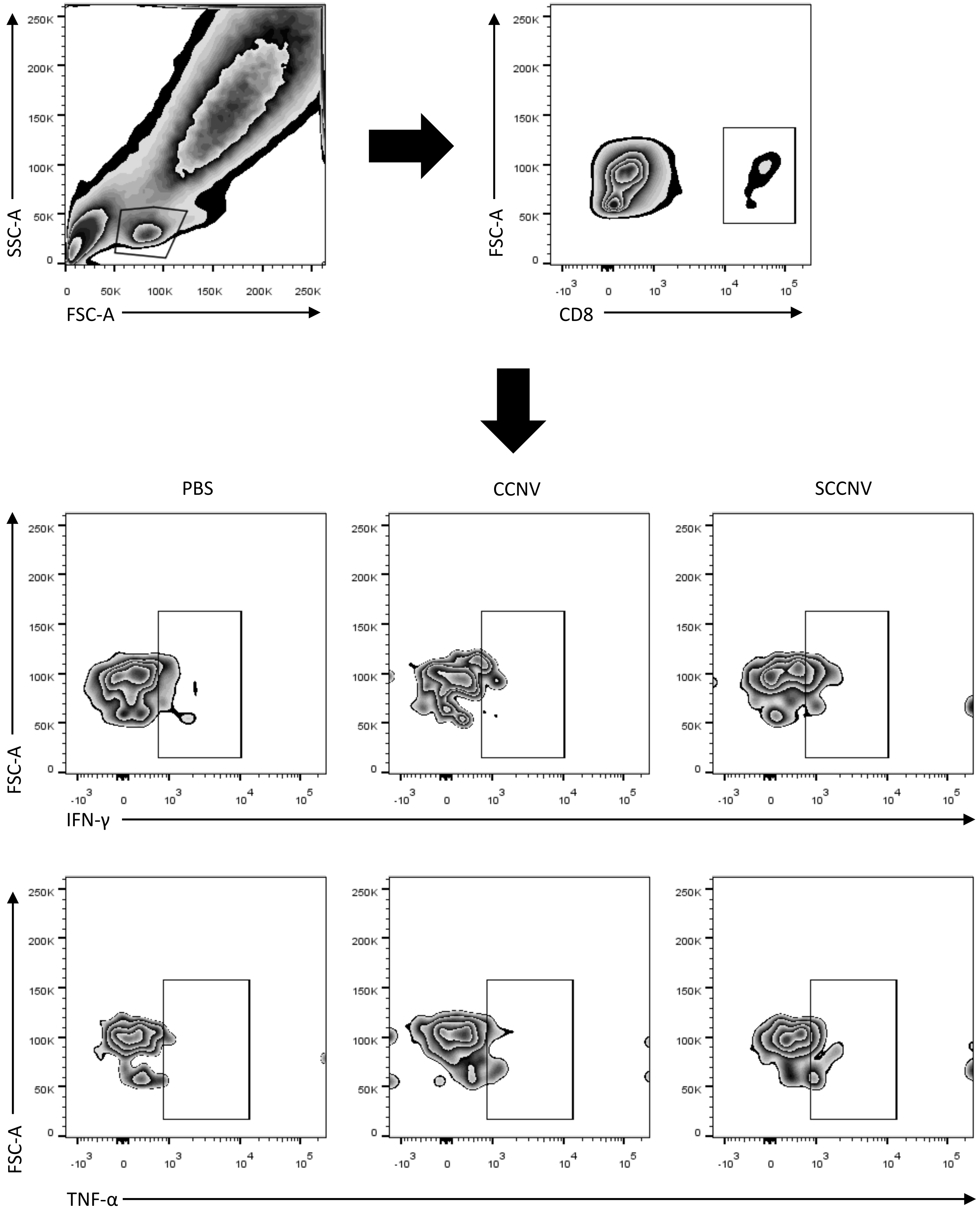


**Supplementary Fig. 10.** Gating strategy of TIL analysis in Figure 6E.

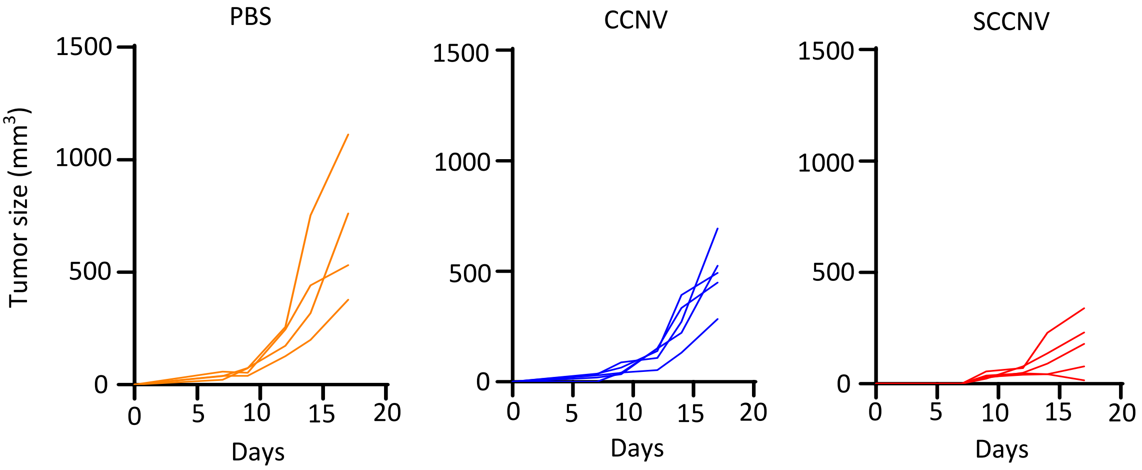


**Supplementary Fig. 11.** Individual tumor growth profiles of B16F10 prophylactic model shown in Figure 6H. n = 5.

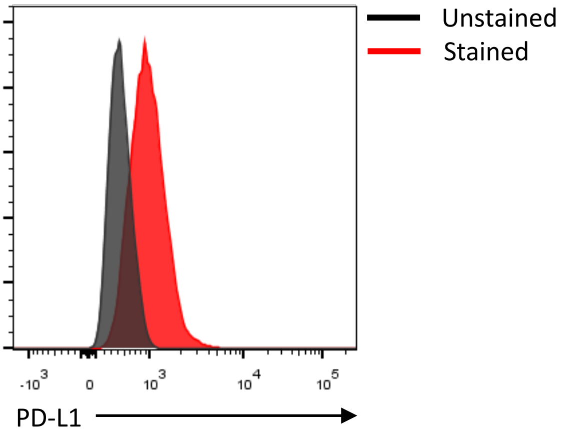


**Supplementary Fig. 12.** FACS analysis of PD-L1 expression of 4T1-Luc cancer cells used in metastatic tumor model in Figure 7.

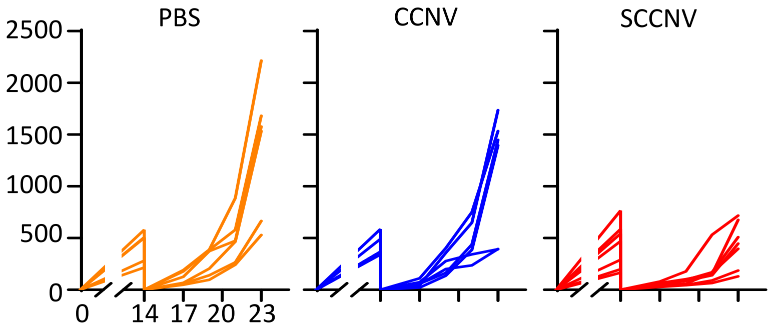


**Supplementary Fig. 13.** Individual tumor growth profiles of post-surgery recurrence of B16F10 tumor model shown in Figure 8B. n = 8.
